# Large environmental changes reduce valence-dependent belief updating

**DOI:** 10.1038/s41598-024-61207-y

**Published:** 2024-05-07

**Authors:** Juan Cruz Beron, Guillermo Solovey, Ignacio A. Ferrelli, María E. Pedreira, Rodrigo S. Fernández

**Affiliations:** 1 Instituto de Fisiología, Biología Molecular y Neurociencias (IFIByNE)-CONICET, Buenos Aires, Argentina; 2 Facultad de Ciencias Exactas y Naturales, Universidad de Buenos Aires, Buenos Aires, Argentina; 3 Facultad de Ciencias Exactas y Naturales, Instituto de Cálculo, UBA-CONICET, Buenos Aires, Argentina; 4 Laboratorio de Neurociencia de la Memoria, Facultad de Ciencias Exactas y Naturales, Universidad de Buenos Aires IFIByNE, CONICET, Ciudad Universitaria (C1428EHA), Buenos Aires, Argentina

**Keywords:** Belief updating, COVID-19, Threat-anxiety, Computational modeling, Optimism bias, Human behaviour, Decision

## Abstract

When updating beliefs, humans tend to integrate more desirable information than undesirable information. In stable environments (low uncertainty and high predictability), this asymmetry favors motivation towards action and perceived self-efficacy. However, in changing environments (high uncertainty and low predictability), this process can lead to risk underestimation and increase unwanted costs. Here, we examine how people (n = 388) integrate threatening information during an abrupt environmental change (mandatory quarantine during the COVID-19 pandemic). Given that anxiety levels are associated with the magnitude of the updating belief asymmetry; we explore its relationship during this particular context. We report a significant reduction in asymmetrical belief updating during a large environmental change as individuals integrated desirable and undesirable information to the same extent. Moreover, this result was supported by computational modeling of the belief update task. However, we found that the reduction in asymmetrical belief updating was not homogeneous among people with different levels of Trait-anxiety. Individuals with higher levels of Trait-anxiety maintained a valence-dependent updating, as it occurs in stable environments. On the other hand, updating behavior was not associated with acute anxiety (State-Anxiety), health concerns (Health-Anxiety), or having positive expectations (Trait-Optimism). These results suggest that highly uncertain environments can generate adaptive changes in information integration. At the same time, it reveals the vulnerabilities of individuals with higher levels of anxiety to adapt the way they learn.

## Introduction

Asymmetrical belief updating refers to the phenomenon where desirable information is more readily incorporated than undesirable information^1,2^. This pattern-also called “optimism bias”- has been observed in various domains such as economic prospects, mental health, climate change, and cognitive abilities^3–10^, and it is primarily attributed to a valence effect, where better-than-expected information is more readily integrated than worse-than-expected information^11,12^. The presence of an asymmetry in the updating of beliefs could be indicative of good mental and physical health^13,14^. When people suffer from a mental health disorder or the context demands increase (such as stress), valence-dependent information processing is sensitive to these changes^9,10,15,16^.

People derive utility from beliefs to the extent that holding certain beliefs about the self, the world, or the future may have hedonic or instrumental (rewarding) properties^10,17–19^. Maintaining positive beliefs can be inherently rewarding, fostering a sense of self-competence and personal growth. From a rational standpoint, one might consider that optimal decisions are made with more accurate and realistic information. However, maintaining positive expectations about the self and others can serve an adaptive function, as it is associated with increased self-efficacy, positive emotions, reduced stress, and a sense of control over outcomes^4,20–22^. In relatively stable environments (low uncertainty and high predictability), these expectations can help reduce risk aversion, motivate action, and encourage exploration of new possibilities. However, overconfidence and over-optimism are associated with negative outcomes both at the individual level, such as work underperformance or misconduct, and at the social level, including severe disasters and medical decision-making errors^1,23,24^.

Our ability to learn from the world and integrate new information is adjusted according to the environmental/contextual demands and our affective state^25–27^. Faced with an acute threat, belief updating becomes more balanced, and individuals seek more information to reduce uncertainty, as the cost of neglecting undesirable information (risk underestimation) might be life-threatening^15,24,27^. For instance, Garrett et al.^15^ found that when individuals are stressed before engaging in a belief updating task, the incorporation of desirable and undesirable information becomes more balanced (symmetric). Moreover, they found the same pattern in firefighters on duty who are commonly confronted with acute stress and demanding contexts. Factors such as personality traits or psychiatric history can also influence how individuals incorporate information into their belief systems^6,8,10,16^. For example, in a previous report, we found that higher levels of Trait-Anxiety were linked to a greater tendency to update beliefs based on desirable information, while the impact of negative or undesirable information was relatively minimal^16^. As anxiety is defined by threat overestimation, we hypothesized that individuals with higher levels of Trait-Anxiety tend to experience more relief when they overestimate the threat of a negative event and subsequently receive desirable information that contradicts their initial estimate^28,29^.

Large environmental changes characterized by unpredictability and high uncertainty, such as the COVID-19 pandemic, induce stress and negative affect which influence information seeking and integration^26,30–34^. For example, a study found that the COVID-19 pandemic increased paranoia symptoms and altered belief updating^35^. Another report found that the pandemic increased anxiety symptoms and amplified the tendency to seek information to reduce uncertainty^30^. Initial mandatory quarantine during the COVID-19 pandemic was linked to worsened mental health and increased psychological distress^31,36^. Argentina, in particular, had one of the strictest and longest lockdowns in the world^36^. At the time the number of cases grew exponentially worldwide, no treatment or vaccine was available and the lethality of the virus SARS-COV2 was unknown. This provided an ecological context to study belief updating during an abrupt environmental change associated with high uncertainty and low predictability. Here we study how people with different levels of anxiety update beliefs and seek information about COVID-19 in a highly uncertain and unpredictable context such as the COVID-19 quarantine. To this end, 17 days after the beginning of Argentinian mandatory quarantine and 33 after the first case of COVID-19 reported in the country, subjects reported their media consumption, completed different anxiety scales, and performed an adapted belief update task. To improve the ecological inference of the study, we used stimuli related to the stressor and the cause of the contextual change: the SARS-COV2 virus. In addition, we complemented this approach using validated computational models of the task^12^ to test our hypothesis.

From the above, we predicted that the quarantine context would be associated with a more balanced information integration as it represents an uncertain and unpredictable threat. Moreover, highly anxious individuals would exhibit asymmetrical belief reflecting a difficulty in adapting the integration of information to an environment that changed abruptly. Finally, we expected levels of high anxiety to be positively associated with information seeking about COVID-19.

## Methods

### Participants

A total of 388 participants (235 females, 150 males, and 3 non-binary) from Buenos Aires (Argentina) with a mean age of M = 38.28 + /– SEM = 0.13, range 18–70 years old, took part in the study (Supplementary Material S1). Participants were recruited using social media, institutional emails, and announcements. The study was conducted online via a R Shiny app. Data was collected 17 days after the beginning of the mandatory quarantine for 10 days, between April 6th to April 16th, 2020. By then, there were approximately 3000 confirmed cases of COVID-19 in Argentina and 145 deaths from the disease. Before the experiment, participants completed self-reported trait-measures: The Trait subscale of the State-Trait anxiety inventory^37^, the short form of state anxiety inventory^38^*,* the short health anxiety inventory (SHAI)^39^, and the life orientation test-revised^40^. Finally, socio-demographic information such as age, gender, and education were also collected. All procedures contributing to this work comply with the ethical standards of the relevant national and institutional committees on human experimentation and with the Helsinki Declaration of 1975, as revised in 2008. Furthermore, all procedures were approved by the Lanari ethical committee. Online informed consent was obtained from all subjects.

### Stimuli

Participants were presented with 24 facts related to the COVID-19 pandemic about the contagion risk (Supplementary Material S2; i.e. *On average, how many days after coronavirus (SARS-CoV-2) infection do the first symptoms appear?*), speed of transmission, the state of the healthcare system and the risk to the general population (i.e. *What percentage of cases require intensive therapy and mechanical ventilation?*). The average probability and frequency of each stimulus were obtained from public national sources (https://www.argentina.gob.ar/salud) and international news agencies (Our World in data https://ourworldindata.org/coronavirus, and the Coronavirus Resource Center of the Johns Hopkins University, https://coronavirus.jhu.edu/map.html).

### Measures

#### COVID-19 related information seeking

Participants answered how much time they spend consuming media (internet, TV, radio, etc.) related to the COVID-19 pandemic on a Likert scale from 1 to 5 being 1 “less than one hour daily” to five being “more than 8 h”. They also rated the importance (relevance) they gave to the information provided using the same scale where 1 was “Nothing at all” and 5 was “Very important”.

### Self-report measures

*Spielberger state and trait anxiety inventory* (STAI-T; Ref.^37^)*. *We used the Trait subscale to measure Trait anxiety. The STAI-T consists of a 20-item scale that assesses the level of anxiety as a general stable trait. Our sample scored M = 22.46 + /– SEM = 0.52 and range = 3–54.

*The short form of state anxiety inventory* (sSTAI-S; Ref.^38^). The sSTAI-S is a 6 item likert scale that assesses the current overall level of anxiety. The sample scored M = 14.52 + /- SEM = 0.35 and range = 0–46.

*Short health anxiety inventory* (SHAI, Ref.^39^). The SHAI is an 18 item self-report questionnaire designed to assess health anxiety in individuals. We obtained a 5.26 + /– SEM = 0.13 and range = 0–11.

*The life orientation test-revised* (LOT-R; Ref.^40^) was used to assess Trait-Optimism and expectations regarding positive outcomes in life. It consists of 10 items and respondents rate their level of agreement or disagreement using a Likert scale. The mean score was M = 21.45 + /– SEM = 0.19 and range = 9–30.

### Belief update task

**Figure 1:**
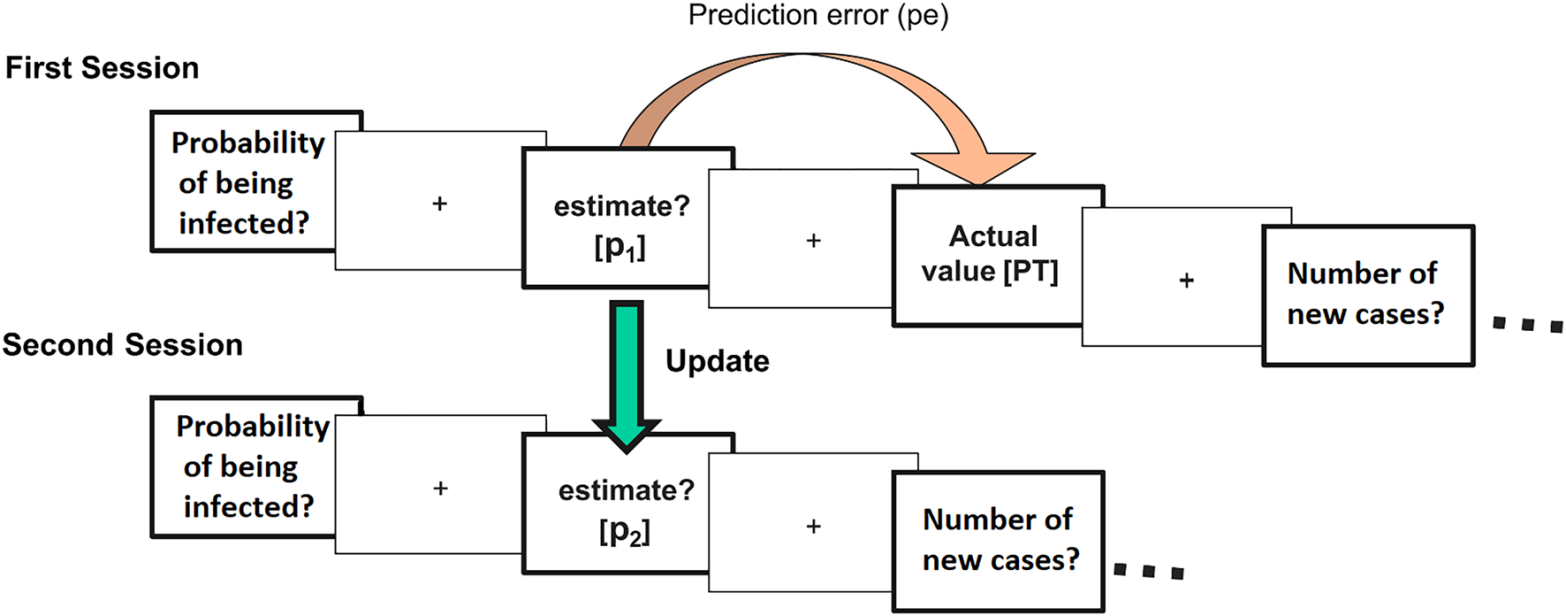
Belief update task. On each trial, subjects were asked to estimate the probability or frequency of a COVID-19 pandemic related fact (First estimate – P1, First Session). Then the actual probability or frequency of the event was presented (True Value – PT). Trials were classified according to their valence. When P1 < PT it was considered that the subject received desirable information (“good news”). On the other hand, when P1 > PT the trial was classified as undesirable information (“bad news”). After finishing the 24 trials (events), participants proceeded to the second round in which they were again asked about the probability or frequency of the same facts in a new random order (P2). The update parameter is calculated as the absolute difference between the participants' estimates in the two sessions (P2-P1), before and after the information (PT) was given.

Participants performed an online belief update task depicted in Fig. 1. In a first round of questions, each trial involved a probability or frequency estimation (First estimate—P1) related to the COVID-19 pandemic facts, such as the probability of being infected, number of new cases, or number of available beds in the healthcare system. Participants had 5 s to respond with a slide, after which the actual probability or frequency of the event (PT) was presented for 2 s. Once the 24 trials were completed the second round of questions began. Participants were presented again with the same questions and asked to re-estimate (Second estimate—P2) the probability/frequency of them.

### Data analysis

Values from media consumption and relevance were used to create an information seeking variable, estimated as the average between those two variables. Information seeking was then entered in a linear regression using state-trait measures and demographics as predictors. The model was specified as follows:

$$ \begin{aligned} {\text{Information Seeking }} & = \beta 0 \, + \beta 1 \, Trait - Optimism \, + \beta 2 \, Trait - Anxiety \, + \beta 3 \, Health - Anxiety \, \\ & \quad + \beta 4 \, State - Anxiety \,  + \beta 5 \, Age \, + \beta 6 \, Gender \, + \beta 7 \, Education \, + \varepsilon , \end{aligned} $$

where β0 represents the intercept, β1 to β7 stands for the coefficients of each predictor variable, and ε represents the error term.

Update was defined as the difference between the subject´s first estimate (P1) and second estimate (P2). Prediction error (PE) was defined as the absolute difference between P1 and the true value (PT) for that fact. Valence classification (desirable or undesirable) of each trial was performed according to the difference between subjects initial estimate (P1) and the true value (PT). If the true value (PT) for a stimulus was lower than the participant's initial estimate (P1 > PT), it was considered desirable information (“good news”). Conversely, a trial was classified as undesirable information (“bad news”) if the true value (PT) was higher than the subject´s initial estimation (P1). Trials with zero PE were excluded from analysis as they cannot be classified according to their valence. Before data analysis subject´s estimated probabilities and frequencies were transformed and normalized between 0 and 1.

We used a single linear mixed-effects (hierarchical) model to fit the data^41,42^. We included random intercepts for subject and stimuli, to account for potential clustering of observations. We defined a model which explained belief update as a function of task predictors (Valence, PE, and PT), state-trait measures (Optimism, State-Anxiety, Health-Anxiety and Trait-Anxiety), information seeking and socio-demographic information^8,16,43^:

$$ \begin{aligned} {\text{Update }} & = \beta 0 \, + \beta 1 \, Valence \, + \beta 2 \, PE \, + \beta 3 \, PT \, + \beta 4 \, Trait - Anxiety \, + \beta 5 \, Health - Anxiety \, \\ &\quad+ \beta 6 \, State - Anxiety \, + \beta 7 \, Trait - \, Optimism \, + \beta 8 \, Age \, + \beta 9 \, Gender \, \\ &\quad + \beta 10 \, Education \, + \beta 11 \, Information \, Seeking \, + \beta 12 \, \left( {Trait - Anxiety \, \times \, Valence} \right) \, \\ &\quad + \beta 13 \, \left( {Health - Anxiety \, \times \, Valence} \right) \, + \beta 14 \, \left( {State - Anxiety \, \times \, Valence} \right) \, \\ &\quad + \beta 15 \, \left( {Trait - Optimism \, \times \, Valence} \right) \, + \, u0 \, \left( {1 \, + \, Valence \, | \, Subject} \right) \,  + \, u1 \, \left( {1| \, Fact} \right) \, + \varepsilon . \end{aligned} $$

where β0 to β15 represent the fixed effects coefficients for each predictor variable and interaction term. U0 and u1 represent the random intercepts and varying slope (Valence) for subject and fact. Lastly, ε represents the error term. We fitted the model using the *lmer* () function from *lme4* in R. In this report, we present the coefficients (β) and its 95% confidence intervals (CI) extracted from the statistical model (treatment coding) and the average marginal effects (M) to test the effects of interest.

### Computational model of belief update task

We implemented a generic reinforcement learning model^44^ of the belief update task adapted from previous work^12,45^. The model assumes that belief updating is proportional to the prediction error (PE) weighted by a learning rate (LR) parameter (Supplementary Material S3).

$$ {\text{Belief Update }} = {\text{ PE }} \times {\text{ LR}}. $$

The LR indicates the tendency of each subject to update their beliefs in response to the size of PE. To test the update bias (desirable information > undesirable information), LR is estimated separately for desirable and undesirable information.

$$ {\text{LR}}_{{{\text{desirable}}}} = {\text{ Alpha }} + {\text{ Asymmetry}}, $$

$$ {\text{LR}}_{{{\text{undesirable}}}} = {\text{ Alpha }} - {\text{ Asymmetry}}. $$

The Alpha component represents the tendency to learn from errors regardless of the valence of information (desirable/undesirable). An Alpha value of 1 implies that the update is precisely equivalent to the PE, while an Alpha value less than 1 suggests updates that are smaller than the PE. On the other hand, the Asymmetry component indicates how much updating is biased by the valence of the PE. An Asymmetry parameter with a value of 0 indicates equal learning rates for both desirable and undesirable information. Conversely, an Asymmetry value different from zero suggests that the LR for desirable and undesirable information is different (biased).

Parameter necessity was tested using model comparison following previous reports^12,43,45^. To test biased belief updating, the full model (m1) was compared against a version (m2) with Alpha as a free parameter and Asymmetry priors variance fixed to zero. Additionally, to test whether belief updating was solely driven by PE, m1 was compared against a model (m3) with Alpha prior variance fixed to zero and a free Asymmetry parameter. Simulation of each model prediction is in Supplementary Material S3. All models demonstrated convergence, as indicated by the Gelman-Rubin r statistic (r < 1.01). These models were fitted using four chains consisting of 4000 iterations and 2000 warm-up iterations. Model comparison was based on the Leave-One-Out Information Criterion (LOOIC) of the *loo* package in R. A smaller LOOIC value suggests a better fit for the model. Lastly, model validation was performed using a Posterior Predictive Check (PPC) by comparing model predictions of the model with observed behavioral data.

### Consent statement

All subjects provided written informed consent.

## Results

### Behavioral and modeling results of the belief update task during the COVID-19 quarantine

**Figure 2:**
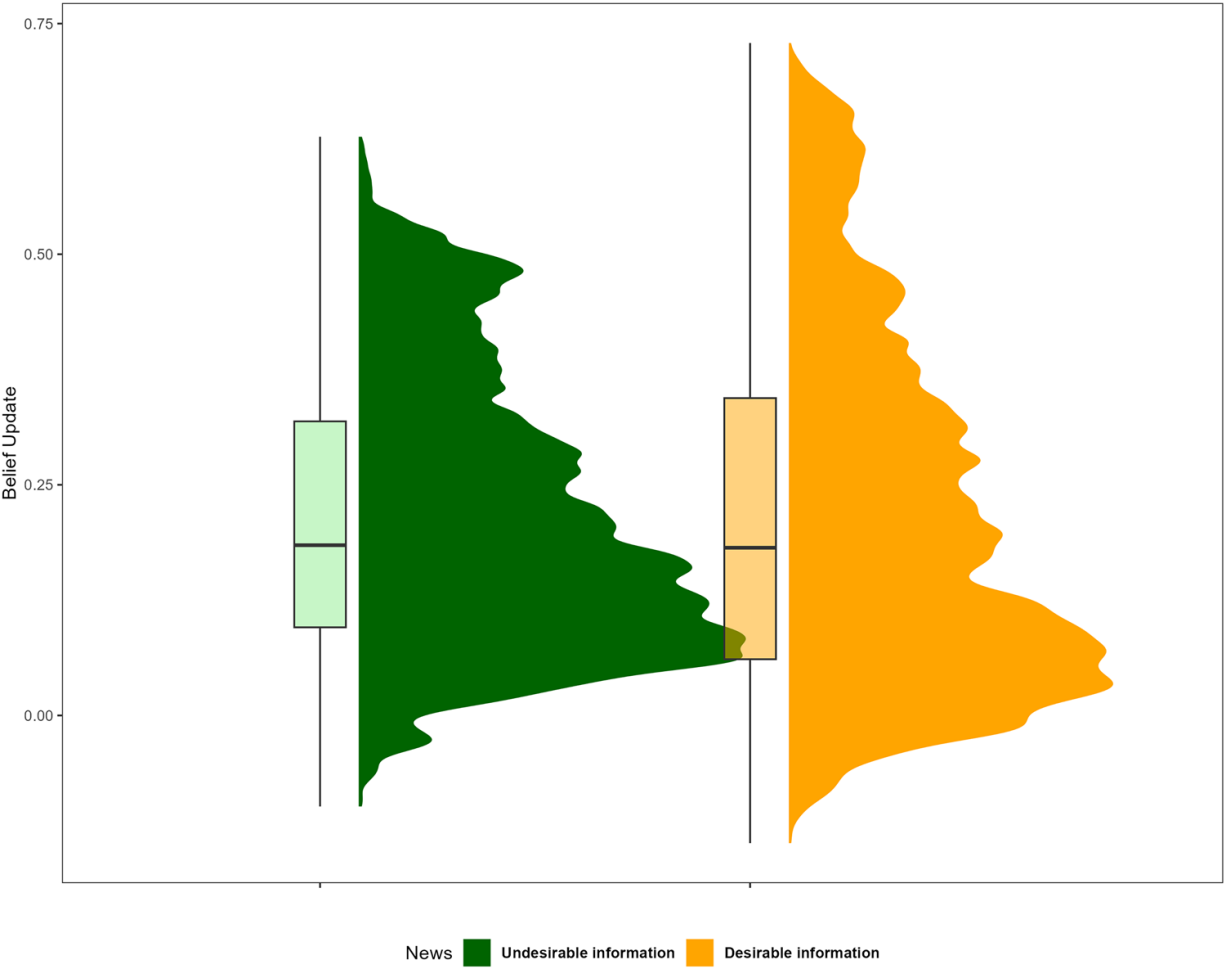
Belief update results. Marginal means. Effect of valence (desirable/undesirable information) in the belief update task during the COVID-19 quarantine. Asymmetrical belief updating was eliminated: desirable and undesirable information were integrated to the same extent.

To analyze belief updating we implemented a single hierarchical linear regression where the subject’s update was explained by the news valence (desirable/undesirable information), the prediction error (PE), the true value of the stimuli (PT), and the Trait-State measures. Our results indicate that asymmetrical belief updating was eliminated during the COVID-19 quarantine. Specifically, desirable (M = 0.218 [0.211, 0.226]) and undesirable (M = 0.216 [0.209, 0.223]) information were integrated to the same extent (Fig. 2, β = − 0.07 [− 0.06, 0.09], p > 0.05) suggesting that valence-dependent belief updating was abolished during a large and abrupt environmental change. As expected, greater values of PE predicted an increase in the belief update (β = 0.878 [0.857, 0.899], p < 0.001). However, the true value (PT) of each fact was not associated with update behavior (β = − 0.04 [− 0.11, 0.04], p > 0.05). Interestingly, COVID-19 related information seeking, was positively associated with belief update (β = 0.008 [0.001, 0.015] p = 0.036), suggesting that more media consumption was associated with increments in update behavior. Similarly, demographic characteristics influenced the degree to which beliefs were updated. In this sense, higher levels of education correlated positively with belief update (Middle Education Level β = 0.024 [0.006, 0.042], p = 0.010; High Education Level β = − 0.023 [0.004, 0.042], p = 0.017) while Age showed the opposite (β = − 0.001 [− 0.001, − 0.000], p = 0.005).

Computational modeling supported these results and provided complementary evidence for the absence of valence-dependent belief updating during the mandatory COVID-19 quarantine. Model comparison indicated that the model that assumed balanced (symmetrical) belief updating (m2 model, LR = Alpha) provided a better model fit (LOOIC = 17,008.9, SE = 11.2; WAIC 17,072.1, SE = 10.1) than the other two models that assumed asymmetrical update driven by PE and information valence (model m1 LR = Alpha + Asymmetry, LOOIC = 17,560.5, SE = 13.2; WAIC = 17,429.1, SE = 11.8) or solely information valence (m3 model LR = Asymmetry, LOOIC = 17,438.8, SE = 10.8, WAIC = 17,246.5, SE = 10.5). The average Alpha parameters of the winning model m2 were significantly smaller than 1 (M_Alpha_ = 0.93 [0.92, 0.94], T (373) =− 15.61, p < 0.001), showing that update behavior was not entirely driven by PE size. To determine the effectiveness of the fitted Reinforcement Learning model parameters (m2) in producing meaningful predictions, we conducted a model validation using PPC (Supplementary Fig. S4). Comparison between predicted update behavior and observed data indicates that the model accurately reproduces the data (r = 0.91, p = 0.001), and supports the winning-model.

### Association between state-trait measures and belief updating

**Figure 3:**
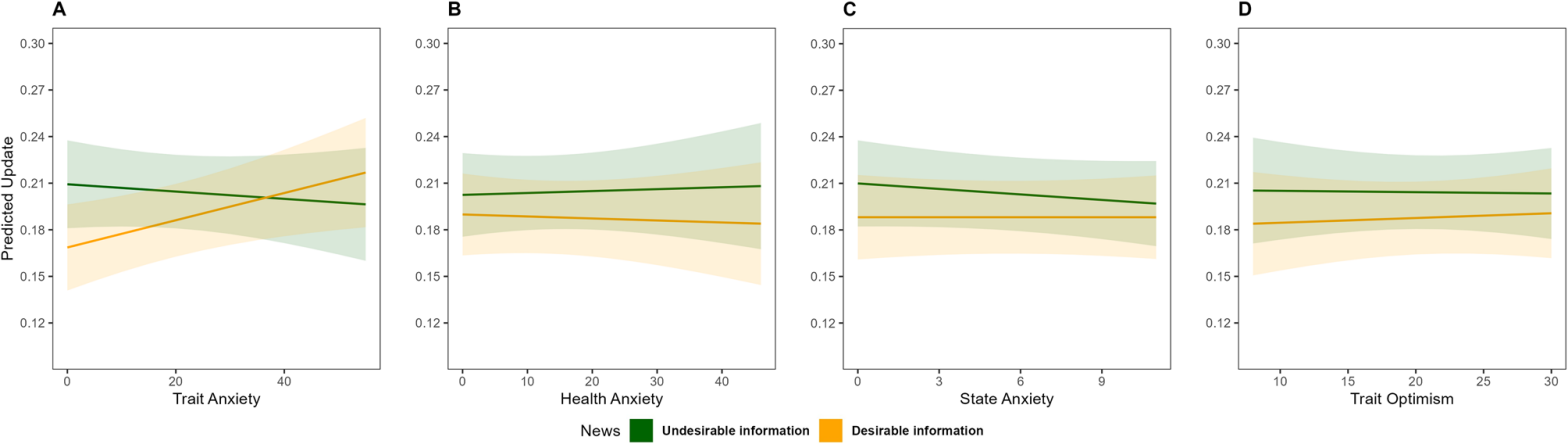
Belief updating as a function of trait-measures and news valence (desirable/undesirable information). Marginal effect (predicted update) of Trait-measures on update behavior. (**A**) trait-anxiety, (**B**) health-anxiety, (**C**) State-anxiety, and (**D**) Trait-optimism.

In line with our previous report^16^, Trait-Anxiety was related to the belief update asymmetry as information integration varied according to Trait-Anxiety levels and information Valence (Fig. 3A, main effect Trait-Anxiety β = 0.0001 [− 0.0001, 0.0001], p > 0.05; Trait-Anxiety × Valence Interaction β = 0.001 [0.001, 0.002], p = 0.02). Higher Trait-Anxiety levels were associated with an increase in update behavior for desirable information (β = 0.0008 [0.0001, 0.001], p = 0.01) but not for undesirable information (β = − 0.0002 [− 0.0001, 0.0005], p > 0.05). However, we did not find evidence that the integration of information was related to the overall health concerns (Fig. 3B, Health-Anxiety β = 0.0002 [− 0.0001, 0.0003], p > 0.05, Health-Anxiety × Valence Interaction β = − 0.0001 [− 0.0001, 0.0001], p > 0.05), temporary anxiety (Fig. 3C, State-Anxiety β = − 0.0001 [− 0.0001, 0.0001], p > 0.05; State-Anxiety × Valence Interaction β = 0.00001 [− 0.00001, 0.00001], p > 0.05), or positive expectations (Fig. 3D, Trait-Optimism β = − 0.0001 [− 0.0001, 0.0001], p > 0.05, Trait-Optimism × Valence Interaction β = 0.0001 [− 0.0001, 0.0001], p > 0.05). Finally, the Alpha parameter was not associated with State-Trait measures either (Supplementary Fig. S5).

### Information seeking during the COVID-19 quarantine

Regression analysis showed that State-Anxiety and Trait-Anxiety was associated with a self-reported measure of COVID-19 related information seeking during mandatory quarantine. Specifically, higher levels of State-Trait Anxiety were associated with more media consumption and perceived relevance (State-Anxiety β = 0.049 [0.021–0.077], p = 0.001; Trait-Anxiety β = 0.010 [0.002, 0.019], p = 0.019). Furthermore, Trait-Optimism and Health-Anxiety were not associated with COVID-19 related information seeking (Trait-Optimism β = 0.019 [− 0.002, 0.040], p > 0.05; Health Anxiety β = 0.006 [− 0.005, 0.017], p > 0.05). In relation to demographics, age was positively correlated with more COVID-19 information seeking. (β = 0.009 [0.003, 0.014], p = 0.003) and being male with the opposite pattern (β = − 0.144 [− 0.283, − 0.006], p = 0.041).

To elucidate whether the levels of information seeking were associated with more accurate estimates that could affect the interpretations of the results, we modeled the subjects´ initial estimates (P1) using a mixed-effect linear regression. We found that COVID-19 related information seeking seemed to inflate subjects initial estimates (P1; β = 0.015 [0.005, 0.025] p = 0.004). However, COVID-19 related information seeking was not associated with more accurate responses such as lower levels of PE (r = − 0.02 [− 0.04, 0.0001], p > 0.05). State-Trait Anxiety and Optimism were not related to initial estimates (P1, p > 0.05).

## Discussion

Belief updating cannot be reduced to an automatic cognitive process as motivational factors and individual trait differences alter information integration^10,12,18^. The current ecological study provides evidence that valence-dependence updating is eliminated after an abrupt and threatening contextual change such as the COVID-19 pandemic. Specifically, we found that when presented with threat-related information about the COVID-19 pandemic, desirable and undesirable information is integrated equally. These findings are also confirmed by computational modeling which established that belief updating can be explained by the prediction error (PE) and a learning rate (LR) without the need for an Asymmetry parameter for desirable and undesirable information. Moreover, we showed that the absence of valence-dependent updating was general across individuals and independent of the temporary changes in anxiety (State-Anxiety), health concerns (Health-Anxiety), or having positive expectations (Trait-Optimism). Previous work in a stable (pre-pandemic) context, established a strong association between Trait-Optimism and the size of asymmetrical belief updating^2,11^. Hence, developed countries were more optimistic about the COVID-19 pandemic trajectory and trusted more their government's ability to resolve the crisis^46,47^. However, it was found that optimism might have been problematic as optimism was related to reduced perceived risk of contagious and lower adherence to protective behaviors^47,48^. Thus, the elimination of the “optimism-bias” and the increments in psychological distress represented an adaptation to environmental threats^1,24,27,31,36^. In this sense, in an uncertain and unpredictable context, the incorporation of undesirable information ("bad news") for a highly optimistic subject, or the integration of desirable information ("good news") for a subject with high health-anxiety may improve decision-making and may carry survival value^29,49^. In a threatening context, the instrumental value of information increases as it improves perceived self-efficacy and reduces uncertainty^22,26,30^. The symmetrical (balanced) integration of information allows better detection of potential threats and consequently improves decision-making by reducing unwanted costs.

Humans learn differently in stable environments (low uncertainty and high predictability) than in volatile environments (high uncertainty and low predictability; Refs.^26,50^). Computational studies suggest that in stable environments it is more adaptive to use a moderate learning rate that allows statistical regularities to be inferred progressively. On the other hand, in volatile environments, a higher learning rate allows us to learn more precisely from recent or more informative events. Most people are able to adjust their learning rate in accordance with contextual changes. However, highly anxious people have deficits in inferring the statistical structure of the world and adapting the learning rate to the demands of the environment^28,49,50^. Additionally, people with higher levels of anxiety have difficulties making decisions in risky or ambiguous situations which leads to sup-optimal decisions^49,51,52^. Notably, in accordance with previous work^16^ we found that Trait-Anxiety was associated with asymmetrical belief updating. In consequence, highly anxious individuals, integrated to a greater extent desirable information relative to undesirable information indicating problems learning in changing environments. Anxiety is defined by threat overestimation, a general tendency to experience negative affect, and avoidance of negative emotional changes (contrasts; Ref.^28,29^). That is, people with higher levels of anxiety prepare and anticipate the worst scenario to regulate its emotional impact. In this sense, it can be thought that with this strategy the likelihood of experiencing “better-than-expected” (relief) outcomes are more frequent. Recent studies indicate that when faced with a stressor, people can make a positive shift in beliefs to recover from stress^53^. Given that people with higher levels of anxiety experience stress and negative affect more frequently and intensely, it could be understood here that people with higher levels of anxiety incorporate more desirable information to regulate negative affect^28,29,51^.

In an effort to make the world more predictable, COVID-19 related information seeking may have served as a strategy to reduce uncertainty and in consequence negative affect^26,28,30,50^. In particular, we found that State-Trait Anxiety was associated with more COVID-19 related Information-Seeking. However, this behavior did not imply more accurate predictions or factual knowledge about the COVID-19 pandemic. The COVID-19 pandemic was associated with belief polarization, political distrust, anti-intellectualism, and conspiracy theories^54^. A limitation of this work was not having included a measure of confidence/trust in the information presented as true value (PT), which could have caused people to reject the data. In addition, this work did not evaluate memory for COVID-19 facts as is commonly done in belief updating studies. This addition could have helped us rule out the possibility that the lack of asymmetry in belief updating could be an interaction effect between PE and memory. Finally, this work could have benefited from a post-COVID pandemic replication to demonstrate that asymmetry in belief updating exists in stable times/environments.

The COVID-19 pandemic produced large changes in our everyday life and contributed to an increase in mental health problems^31,33,36^. This work provides evidence that the belief updating process is sensitive to the valence of information, individual differences (traits), and more importantly, environmental demands. Here, we found that belief updating in the face of an abrupt and uncertain context change such as the COVID-19 pandemic, eliminated asymmetrical updating and led to information integration independent of valence. Furthermore, we reveal this effect using facts directly related to the COVID-19 pandemic and the causes of the quarantine. Finally, we revealed that people with higher levels of anxiety appeared to be insensitive to the change in context as they continued to update COVID-19 related beliefs asymmetrically, supporting the hypothesis that anxious individuals have problems learning in changing environments.

### Supplementary Information

Supplementary Information.

## Data Availability

The data that support the findings of this study are available here: https://osf.io/dyzmr/.
