# Supplementary material for: Large environmental changes reduce valence-dependent belief updating
Source: Sci Rep. 2024 May 7;14:10429. doi: 10.1038/s41598-024-61207-y (PMC11076288; doi:10.1038/s41598-024-61207-y)

**Large environmental changes reduce valence-dependent belief updating**

Juan Cruz Beron^a-b^, Guillermo Solovey^c^, Ignacio A. Ferrelli ^a-b^, María E. Pedreira^a-b^, Rodrigo S. Fernández^a-b^

a-Instituto de Fisiología, Biología Molecular y Neurociencias (IFIByNE)-CONICET, Argentina.

b-Facultad de Ciencias Exactas y Naturales, Universidad de Buenos Aires, Argentina.

c-Instituto de Cálculo, Facultad de Ciencias Exactas y Naturales, UBA-CONICET, Buenos Aires, Argentina.

**Supplementary Material S1.**

**Socio-demographic Table.**

| **Demographics Variables** | | **Mean / n** | **se / Percentage** |
| --- | --- | --- | --- |
| **Age** |  | 38.2 | 0.6 |
| **Education** |  |  |  |
|  | High School | 26 | 6.95% |
|  | University (Incomplete) | 116 | 31.20% |
|  | University (Complete) | 231 | 61.70% |
| **Gender** |  |  |  |
|  | Female | 235 | 62.80% |
|  | Male | 138 | 37.20% |

**Supplementary Material S2.**

List of questions/facts about COVID-19 and Argentina used in the study.

(The data for each question/fact was updated every day).

1. According to official reports, how many confirmed cases of coronavirus were there in the world as of April 5^th^?"
2. "Of the 195 countries in the world, how many reported at least one person infected with coronavirus until April 5^th^?"
3. "Of all the people who were infected with coronavirus (SARS-CoV-2), what percentage had already recovered as of April 5^th^?"
4. "Until April 5^th^, how many deaths were reported in the world from coronavirus (SARS-CoV-2)?"
5. "On average until April 5^th^: Every how many days does the number of deaths in the world from coronavirus (SARS-CoV-2) double?"
6. "On average, how many days after coronavirus (SARS-CoV-2) infection do the first symptoms appear?"
7. "How many coronavirus testing centers are there currently in the province of Buenos Aires?"
8. "In the cases of coronavirus patients who died, how many days after infection did death occur?"
9. "For how many days is a person infected with coronavirus contagious even in the absence of symptoms (SARS-CoV-2)?"
10. "In how many months is it estimated that a vaccine could be ready and available?"
11. "How many hours, on average, does the coronavirus remain on contagious surfaces?"
12. "What percentage of cases require intensive care and mechanical ventilation?"
13. "What percentage of cases are low intensity without developing complications?"
14. "In what percentage of cases do you have a dry cough during the illness?"
15. "In what percentage of cases do you have fatigue during the illness?"
16. "In what percentage of cases do you have difficulty breathing during the illness?"
17. "What percentage of cases are fatal in people over 80 years old?"
18. "What percentage of new cases are due to infections by other people who do not have symptoms or were not diagnosed?"
19. "How many days did it go from 200,000 coronavirus cases to 300,000?"
20. "What percentage of the Argentine population is at greater risk due to age (over 70 years)?"
21. "According to official estimates from the Ministry of Health, if the quarantine is successful, in the most favorable case, what number of cases is expected for June of this year?"
22. "According to official estimates from the Ministry of Health, if the quarantine is not complied with, in the most unfavorable case, what number of cases is expected for June of this year?"
23. "What number of intensive care beds does Argentina have available in total?"
24. "According to the World Health Organization, as of April 5^th^, what percentage of people infected by the virus die?").

**Supplementary Material and Figure S3.**

*Computational model of belief update task:*

Belief Update = PE * LR

Models varied according to the active component of the learning rate (LR). Alpha represents the tendency to learn from Prediction Error (PE) and Asymmetry how much updating is driven by valence.

**M1 full model:**

LR_desirable_ = Alpha + Asymmetry

LR_undesirable_ = Alpha - Asymmetry

**M2 model:**

LR_desirable_ = Alpha + zero Asymmetry

LR_undesirable_ = Alpha - zero Asymmetry

**M3 model:**

LR_desirable_ = zero Alpha + Asymmetry

LR_undesirable_ = zero Alpha – Asymmetry

**Model simulations and predictions:**


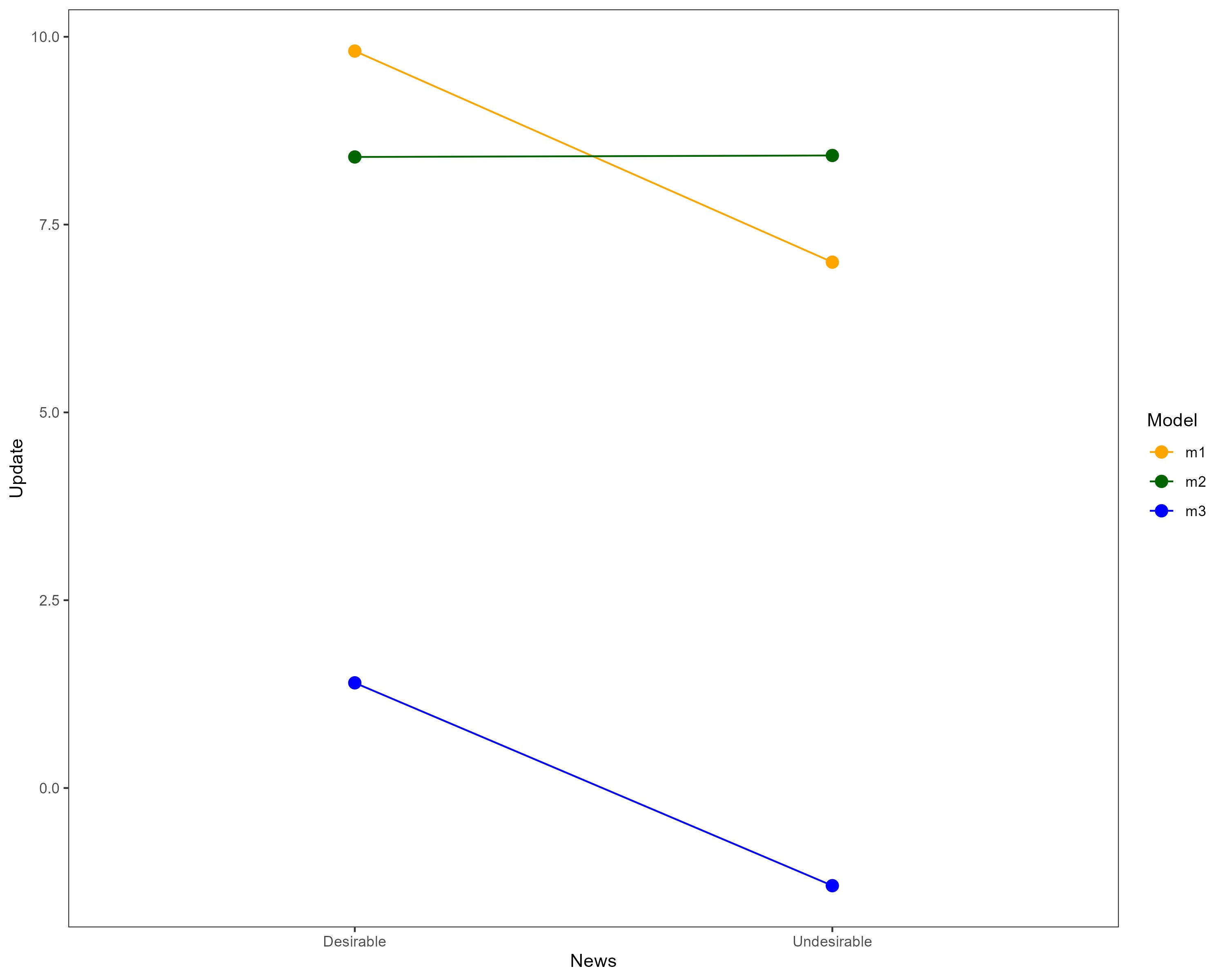


**Supplementary Figure S4.**

Posterior Predictive Check (PPC): Predicted belief updating means based on the winning-model (m2)


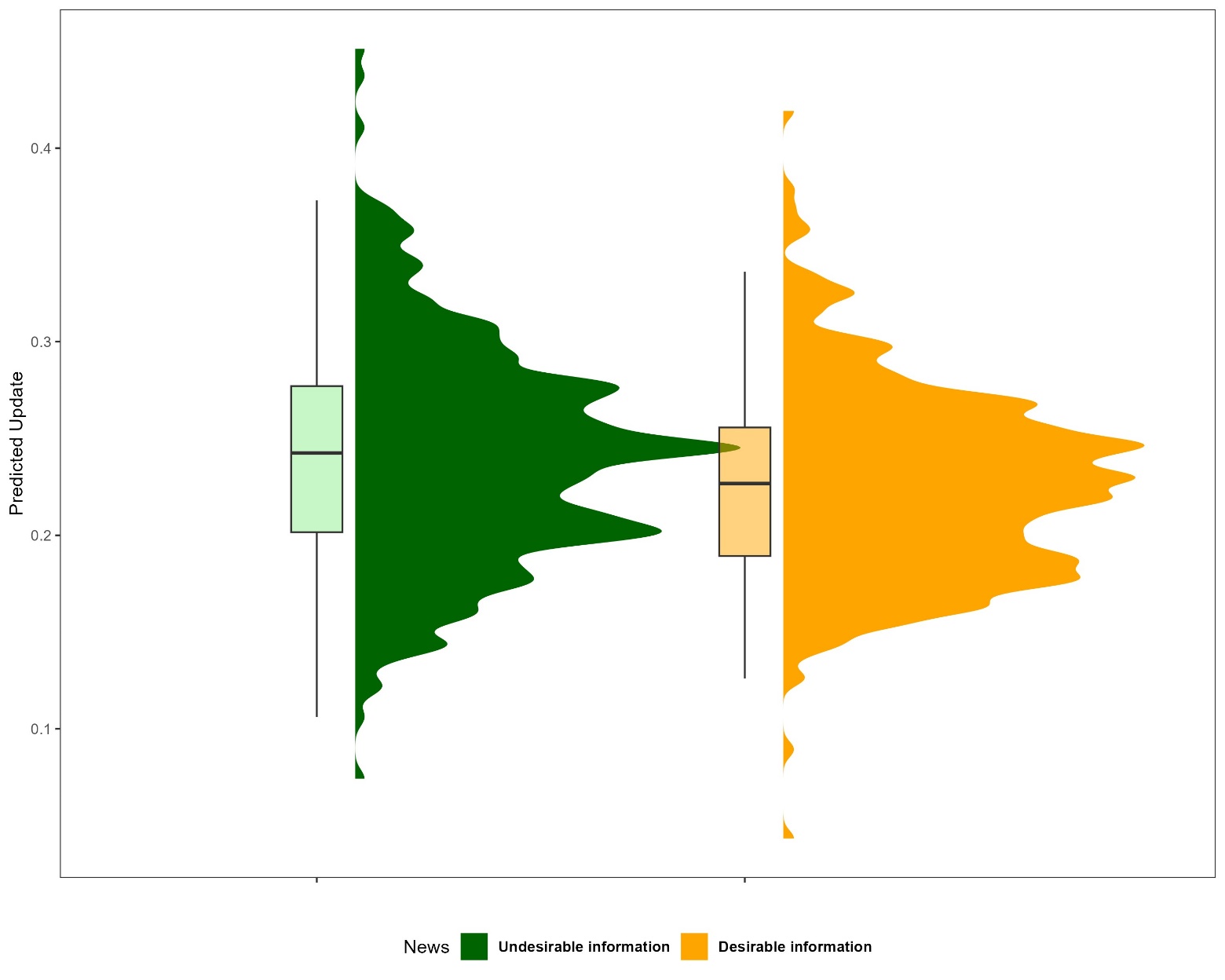


**Supplementary Figure S5.**

- Alpha parameter values of the winning-model m2:


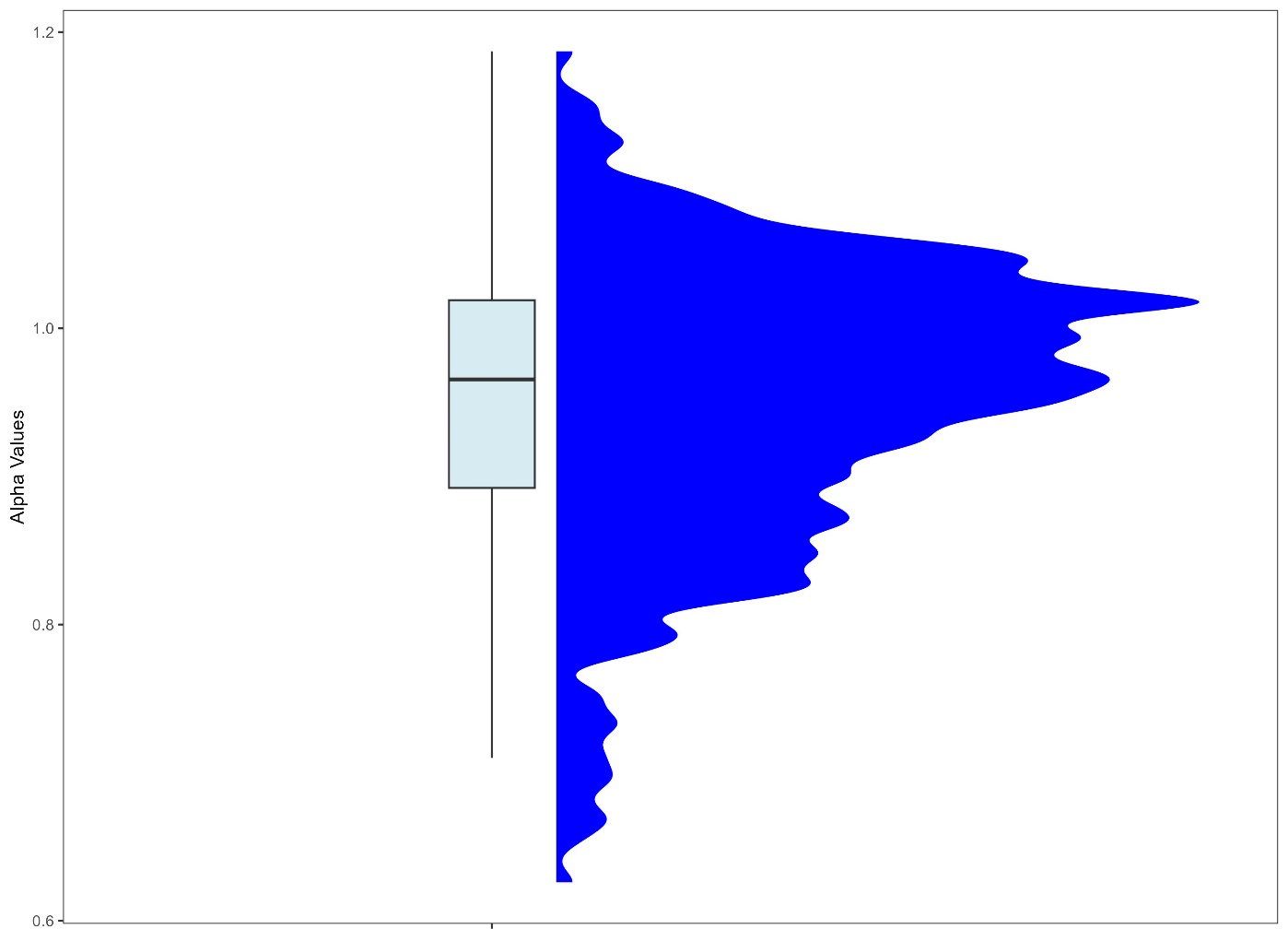


- Association between Alpha parameter and State-Trait measures.


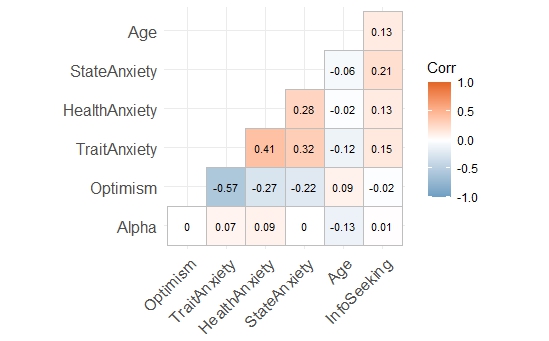

Supplement: Supplementary file 1 — Supplementary Information. [file 41598_2024_61207_MOESM1_ESM.docx]
